# Wasp intestinal cues drive yeast toward outbreeding strategies

**DOI:** 10.1093/ismejo/wraf243

**Published:** 2025-11-01

**Authors:** Silvia Abbà, Liam D Adair, Francesca Barbero, Luca P Casacci, Ilija Dukovski, Francisca Font-Verdera, Tom Hawtrey, Elizabeth J New, Jukkrit Nootem, Pramsak Patawanich, Lukas Patten, Marco Polin, Daniel Segrè, Nian Kee Tan, Irene Stefanini

**Affiliations:** Department of Life Sciences and Systems Biology, University of Turin, Via Accademia Albertina 13, 10123 Turin, Italy; School of Chemistry and the Australian Research Council Centre of Excellence for Innovations in Peptide and Protein Science, Eastern Avenue, The University of Sydney, NSW 2006, Australia; Department of Life Sciences and Systems Biology, University of Turin, Via Accademia Albertina 13, 10123 Turin, Italy; Department of Life Sciences and Systems Biology, University of Turin, Via Accademia Albertina 13, 10123 Turin, Italy; Bioinformatics Program, Faculty of Computing and Data Sciences, Boston University, Boston, Massachusetts, United States; Mediterranean Institute for Advanced Studies, (IMEDEA, CSIC-UIB), C/Miquel Marquès 21, 07190, Esporles, Spain; School of Chemistry and the Australian Research Council Centre of Excellence for Innovations in Peptide and Protein Science, Eastern Avenue, The University of Sydney, NSW 2006, Australia; School of Chemistry and the Australian Research Council Centre of Excellence for Innovations in Peptide and Protein Science, Eastern Avenue, The University of Sydney, NSW 2006, Australia; School of Chemistry and the Australian Research Council Centre of Excellence for Innovations in Peptide and Protein Science, Eastern Avenue, The University of Sydney, NSW 2006, Australia; School of Chemistry and the Australian Research Council Centre of Excellence for Innovations in Peptide and Protein Science, Eastern Avenue, The University of Sydney, NSW 2006, Australia; Bioinformatics Program, Faculty of Computing and Data Sciences, Boston University, Boston, Massachusetts, United States; Mediterranean Institute for Advanced Studies, (IMEDEA, CSIC-UIB), C/Miquel Marquès 21, 07190, Esporles, Spain; Bioinformatics Program, Faculty of Computing and Data Sciences, Boston University, Boston, Massachusetts, United States; School of Chemistry and the Australian Research Council Centre of Excellence for Innovations in Peptide and Protein Science, Eastern Avenue, The University of Sydney, NSW 2006, Australia; Department of Life Sciences and Systems Biology, University of Turin, Via Accademia Albertina 13, 10123 Turin, Italy

**Keywords:** *Saccharomyces cerevisiae*, social wasps, *Polistes* spp, association

## Abstract

*Saccharomyces cerevisiae* relies on social wasps (e.g. *Vespa crabro*, *Polistes* spp.) for dispersal and genetic mixing. Unlike most natural environments, wasp intestines provide conditions that support yeast survival, sporulation, spore germination, and mating. This study explores the mechanisms at the basis of this process by examining the wasp gut environment and yeast responses. Molecular analyses based on yeast deletion collection and transcriptomics showed that yeast sporulates in the crop, spores germinate in the gut, and cells ferment in the gut. The crop and gut differ chemically: the gut has more sugars, a higher pH, and (in workers) greater viscosity. *In vitro* tests confirmed yeast survival in both environments, with faster germination in gut-like conditions. Computational models based on these physicochemical traits matched the experimental results. The data obtained provide fundamental insights into yeast progression towards mating within wasps’ intestines and suggest a possible relation between yeast alcoholic fermentation and wasps’ alcohol tolerance, thereby enhancing our understanding of the *S. cerevisiae*-social wasp association.

## Graphical Abstract

**Graphical Abstract:**
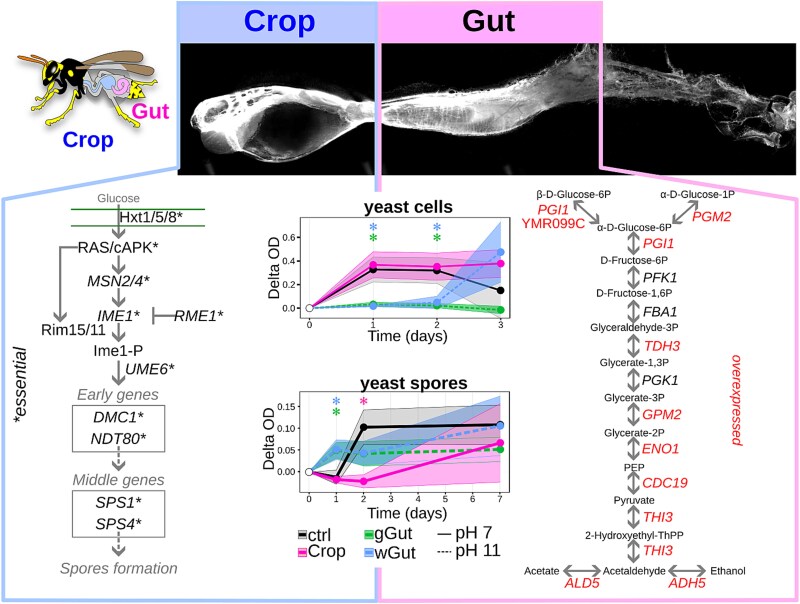

## Introduction

The evolution and ecology of the yeast *S. cerevisiae* have long been considered strictly connected to human activities [1–3]. Nonetheless, the recent isolation of strains from various natural sources has highlighted distinct evolutionary paths between biotechnological and wild strains [4–8]. The spread of natural *S. cerevisiae* strains in wild environments has been associated with the vector and reservoir role of social wasps, such as *V. crabro* and *Polistes* spp. [9]. This association is highly species-specific, as confirmed by studies showing that the intestinal mycobiota of other arthropods rarely included *S. cerevisiae* [10–13]. This yeast-social wasps association, in addition to promoting the dispersal and resilience of *S. cerevisiae* in natural settings, promotes yeast outbreeding (the mating among different yeast strains), a phenomenon otherwise rarely observed in natural environments [14]. To perform outbreeding, natural *S. cerevisiae* strains, mostly diploid, should undergo meiosis (sporulation, to produce haploid gametes) followed by spore germination after elimination of the ascus and release of the contained spores. Sporulation and germination occur in natural settings (e.g. rotting fruits, soil), as a consequence of environmental stresses and nutrient availability fluctuations. Conversely, ascus rupture before spore germination and mating is more complicated to achieve [14]. Natural strains, showing low levels of heterozygosity, are likely the result of inbreeding, the mating among spores deriving from the same parental strain [15]. In contrast, strains isolated from the wasp intestine show high heterozygosity, suggesting outbreeding occurring in this environment, as experimentally proven [14]. Hence, the wasp intestine provides an environmental setting favoring the outbreeding process. Unfortunately, current knowledge of the social wasp intestine is still limited and mostly focused on aspects related to the host physiology, sociality, and evolution [16, 17]. The wasp intestine presents two main compartments likely exposing yeast cells to different and challenging environments: the crop, where a predigestion occurs; and the gut, where digestion is completed and absorption occurs [18–20]. The strict succession of these environments may promote all the steps required for outbreeding through a mechanism i.e. currently unknown. Unraveling the specific association between *S. cerevisiae* and social wasps requires the assessment of the intestinal environment and its impact on yeasts. This study aims to evaluate the physicochemical features of the wasp intestine and examine how these properties influence yeast molecular and physiological responses, thereby promoting inter-strain mating.

## Materials and methods

### Insect collection and rearing

In summer 2024, paper wasps (*Polistes dominula*) were collected using butterfly nets in abandoned fields around Cuneo and Torino (Italy) and then transferred into transparent, clean boxes (29 × 24 × H18.5 cm). The captured wasps were fed with water and sugar under controlled conditions (natural photoperiod and room temperature) until the start of the experiment. In experiments involving wasp feeding with yeast cells, wasps were starved for at least 24 h to ease individual feeding. In this study, we use the term gut to refer to mid- and hindgut.

### Testing yeast outbreeding in the intestine of active wasps and microscopy

Wasps were collected in 2023, transferred to the laboratory, and housed as described in section 2.1 Insect collection and rearing. Before feeding wasps with yeasts, all food resources were removed and, after 72 h of starvation, individual insects were fed with a suspension of two auxotrophic *S. cerevisiae* cells (BY4741 - MATa, his3Δ1 leu2Δ0 met15Δ0 ura3Δ0- and BY4742 -MATα, his3Δ1 leu2Δ0 lys2Δ0 ura3Δ0-) in 50% glucose solution. Twenty-five wasps were fed, each wasp was collected with tweezers, and 10 μL of cell mix (containing 10^8^ yeast cells) was injected directly into its mouth with a pipette. Up to five wasps were analyzed 1, 2, 3, 4, and 8 weeks after feeding; if wasps died naturally in the period between two timepoints, they were excluded from the analysis. Yeast isolates were tested on selective media to identify the strains according to their auxotrophies: the parental strains and the mating product are unable to grow in the absence of histidine, leucine, and uracil; BY4741 in the absence of methionine; and BY4742 in the absence of lysine. To observe the distribution of yeast cells within the intestine, SK1 cells, after an overnight preculture in YPD at 28°C with shaking, were washed twice with sterile water, stained with 1 mm Rose Bengal, and incubated for 10 min at room temperature. After staining, the cells were washed and resuspended in sterile water to a final concentration of 10^8^ yeast cells per 20 μL (the volume individually administered to each wasp). After feeding, wasps were left in individual boxes for 30 min, then killed by freezing, and dissected. The entire intestine was transferred to microscopic slides and visualized with an ECLIPSE Ts2R-FL microscope (Nikon) (excitation: 550 nm, emission: 630 nm).

### Yeast deletion collection: treatment and analysis

The *S. cerevisiae* diploid heterozygous deletion strains pool was used (Thermofisher) [21]. Additional technical information on the deletion collection and its preparation is in the Supplementary Materials. 19 worker wasps, caught in June 2023, were starved for 4 days. Afterward, 10 wasps were fed with 10^8^ yeast deletion pool cells suspended in water (20 μL), with the addition of sugar. 10 aliquots of the deletion collection pool in YPD were maintained in separate sterile tubes in the same condition as the wasps and used as the control for the analysis. Wasps were fed for 4 days with water and sucrose, then killed by freezing and dissected for intestine extraction. The total DNA was extracted from the collected samples by using the DNeasy PowerSoil Pro kit (Qiagen) following the manufacturer’s instructions. Further technical details on the preparation of the sequencing library are reported in the supplementary materials. MiSeq (Illumina) PE 300 sequencing of the purified amplified DNA was performed by Eurofins Genomics. Raw data, deposited in NCBI with the Project ID PRJNA1267698 (BioSample accessions: SAMN48727422-SAMN48727443), were first filtered according to the sequencing quality according to fastqc [22], then aligned with bwa [23] against a custom reference database composed of the Deletion Collection tag sequences, and the number of reads associated with each tag was determined with featureCounts [24]. Differentially represented genes were identified by comparing the abundance of reads in the sample (crop or gut replicate) and in the control with the DESeq function of the DESeq2 R package [25, 26]. Functional enrichment analysis was performed on the list of significant genes (over- or under-represented in crop or gut samples compared to the control) with DAVID [27] against KEGG [28], Reactome [29], and GO [30] databases. Representation of significantly enriched pathways was performed by annotating the schematic representation of the pathways gathered from the corresponding database (KEGG or Reactome).

### Transcriptomic analysis

Worker wasps were caught in May 2024 and reared for at least one week in laboratory conditions (see details in section “Insect collection and rearing”). Five wasps were then individually fed with 10^8^ yeast cells or 10^8^ yeast tetrad, resuspended in a 2% glucose solution in sterile water. Five control wasps received only the glucose solution. Details on the procedures adopted to obtain yeast cells and spores [31] are reported in the supplementary materials. Wasps fed with cells or spores were kept individually for 3 days at 25°C with ad libitum food (glucose solution) availability, then killed in liquid nitrogen and stored at −20°C until dissection. Dissection was performed with sterile tools, crop and gut were separately subjected to RNA extraction with the Trizol procedure following the manufacturer’s protocol, and further processed with the RNeasy Micro Kit (QIAGEN). RNA sequencing was performed after Poly(A)selection (to select eukaryotic mRNA) and sequenced through NovaSeq Xplus paired-end 150 bp sequencing, aiming at 10 M read pairs per sample (Eurofins Genomics). For each sample, reads were aligned against the S288c genome (NBCI RefSeq GCF_000146045.2) with hisat2 [26], and transcript abundances were quantified using featureCounts [24]. Raw data are deposited in NCBI with the Project ID PRJNA1267698 (BioSample accessions: SAMN48727410-SAMN48727421). To exclude signals from the natural wasp gut microbiota, any transcripts identified in control samples were excluded from the transcriptional data of vegetative cells or spores. The genes remaining after this selection were considered expressed in the intestinal compartment if present in at least one sample. Functional enrichment analysis was performed on the list of expressed genes with DAVID [27] against KEGG [28], Reactome [29], and GO [30] databases. Representation of significantly enriched pathways was performed by annotating the schematic representation of the pathways gathered from the corresponding database (KEGG or Reactome).

### Probes calibration and use in the wasp intestines

Probes were synthesized according to literature procedures, with the probes NpRho1, CouPyC6, and BDP_F suitable for pH, viscosity, and saccharides quantification, respectively [32–35]. Probe stock solutions in dimethyl sulfoxide (DMSO) were stored at 4°C in the dark. Detailed technical information on the approach used to prepare the calibration curve is reported in the Supplementary Materials. Briefly, calibration curves were obtained for the probes as follows. The **NpRho1 probe (20 μM), for pH evaluation, was** incubated at room temperature for 30 min with 10 μL of standard pH solutions (4.0, 7.0, and 13.0) in triplicate; deionized water served as blank. Fluorescence was recorded at 580 and 530 nm (excitation 410 nm), and the 580/530 ratio was fitted to pH values. The BDP-F probe (10 μM), for glucose measurement, was mixed with 10 μL of glucose standards (700, 500, 250, 100, and 20 g/l); fluorescence (excitation 496 nm, emission 508 nm) was fitted to glucose concentration. The CouPyC6 probe (0.5 μM) was tested with PEG 4000 in water (500, 350, 250, 125, 62.5, and 0 g/l) to assess viscosity. Additional glucose solutions (800, 500, 220, 70, and 20 g/l) were prepared to evaluate glucose effects on viscosity. Linear and polynomial regressions were performed using the *lm* function in R to fit each calibration curve, and the model withthe *R*^2^ closest to 1 was selected. For each probe test, wasps were reared in laboratory conditions (at least 3 days fed with glucose and water, at room temperature, with natural photoperiod), then wasps were fed a 70% (700 g/l) glucose solution for 2 h, chilled by freezing, and subsequently dissected as previously described [9]. The crop and gut were separated and diluted 1:2 with sterile, deionized water. Each solution was then mixed with the probes (20 μM NpRho1, 0.5 μM CouPyC6, or 10 μM BDP-F), and fluorescence measurement was performed as described above. For each test, a sample with the characteristics of the samples used for the probe calibration was used as a control of the prediction accuracy. The pH, viscosity, and glucose concentrations were calculated according to the formulae obtained with the fitting previously described. All fluorescence readings were performed with a GloMax Discover Microplate Reader instrument (Promega). The amount of alpha-amino nitrogen was assessed with the dedicated Starglass kit, using leucine as a reference standard.

### High-throughput analysis

The wild-type *S. cerevisiae* SK1 strain cells were grown o.n. at 28°C with shaking in YPD. Tetrads (thereafter called spores) were prepared as described for transcriptomics analysis. Cells and spores were dispensed into 96-well plates at a final amount of 2x10^6^ cells or tetrads per well. The tested conditions were all the possible combinations of 2 g/l, 20 g/l, or 200 g/l glucose; 1 mPa*s (crop viscosity), 38 mPa*s (gyne gut viscosity), or 53 mPa*s (worker gut viscosity); 7 or 11 pH (only for cells, corresponding to crop and gut pH); YP (rich medium - YP, 1% yeast extract, 2% peptone), YNB (yeast nitrogen base) with Yeast Synthetic Drop-out without histidine, leucine, tryptophan and uracil, YNB with Yeast Synthetic Drop-out without histidine, leucine, and uracil, and with the addition of 50 mg/l tryptophan. At least three biological replicates were performed for each treatment; a sample without yeast inoculation was used as a control. Samples were incubated at 28°C with mild shaking. The OD600 of each sample was measured right after the inoculation of yeast cells or spores (T0), and subsequently after 1, 2, and 3 days for vegetative cells, and after 1, 2, and 7 days for spores. The measured OD600 values of control samples were subtracted from the OD600 of the corresponding treatments. For each treatment, the delta OD was calculated by subtracting the OD600 at T0 from the OD600 at the measured timepoints. Statistical differences in delta OD between treatments were evaluated through the Wilcoxon-Mann–Whitney test, followed by FDR (false discovery rate) *P* value adjustment. The Yeast Live/Dead kit (Invitrogen) test was used to assess the percentage of alive and dead cells and the number of cells, following the manufacturer’s instructions.

### Dynamic flux balance analysis

Dynamic flux balance analysis (dFBA) was performed using the COMETS platform [36], allowing the stoichiometric modeling of the genome-scale metabolic networks yeast-GEM [37] and iMM904 [38] developed for *S. cerevisiae*. Two computational simulations were performed: one to assess the maximal growth in the absence of a single gene (testing the genes essential or necessary according to the deletion collection experiment results and based on the yeast-GEM metabolic networks, more accurate in terms of genes), the other to evaluate the growth curve and metabolites consumption and production in different environmental conditions (based on the simplified and accurate iMM904 metabolic network). Both models were tested by imposing three different nutrient settings mimicking the optimal growth condition (YPD), the crop content, and the gut content. The scripts used to perform the analysis are available in GitHub, https://github.com/segrelab/waspgut (“Growth_curve.ipynb” to evaluate the growth and metabolites consumption and production; “GENETICS_deletion.ipynb” to perform the gene deletion analysis). Genes excluded in the first model were considered essential for survival in crop or gut settings if resulting in no growth or necessary if the predicted growth was lower than the growth predicted by eliminating the gene in optimal environmental settings (YPD). Functional enrichment analysis was performed on the list of selected genes with DAVID [27] on the GO [30] database. For graphical representation, the ratio of the predicted growth in the tested conditions divided by the predicted growth in the optimal conditions is reported.

## Results and discussion

### Yeast outbreeding occurs in active worker wasps

**Figure 1:**
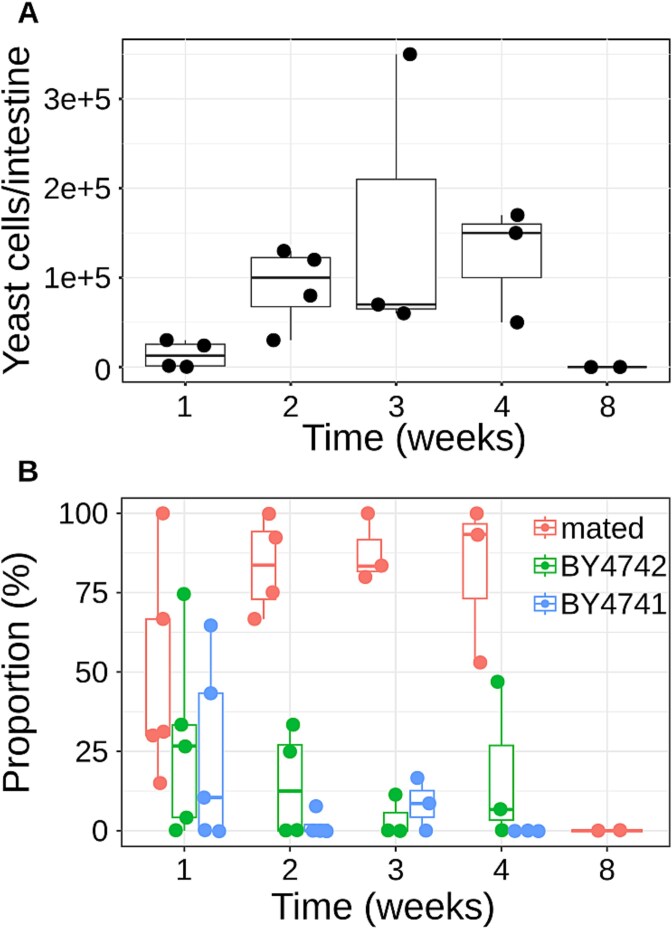
Mated and parental strains percentage in active social wasps fed with auxotrophic strains. The strains BY4741 (MAT a, auxotrophic for methionine, histidine, leucine, and uracil) and BY4742 (MAT alpha, auxotrophic for lysine, histidine, leucine, and uracil) were fed to social wasps. At the indicated time, the wasp intestinal content was cultured in selective medium to isolate the fed strains and their mating product (all requiring histidine, leucine, and uracil to grow). (A) Comparison of the total number of yeast cells isolated from the wasp intestine at the indicated time point. (B) The percentage of the inoculated strains was calculated as the number of isolates requiring also methionine (BY4741) or lysine (BY4742) to survive; the remaining isolates were considered as mating products.

Previous studies have shown intra- and inter-specific yeast cells in *Polistes dominula* gynes’ intestines after a period of hibernation lasting at least three months [14]. No information has been available related to active wasps. By feeding active worker wasps with a mix of haploid strains of the opposite mating type and with complementary auxotrophies (BY4741 - MAT a, auxotrophic for methionine, histidine, leucine, and uracil- and BY4742 - MAT alpha, auxotrophic for lysine, histidine, leucine, and uracil), we quantified yeast cells and assessed the proportions of parental strains and mating products. The number of auxotrophic yeast cells showed an increase until the third week after feeding (Fig. 1A), although the number of cells was not significantly different when compared across multiple timepoints (Wilcoxon-Mann–Whitney FDR > 0.05). Outbreeding products were already observed after the first week after treatment (Fig. 1B). The decline of yeast cells over time in wasp intestines was already observed in the test performed on hibernating wasps [14], and can be ascribed either to a limited capability of yeast cells to survive in this environment, or to the reduced fitness of laboratory-made auxotrophic strains, mostly absent in natural settings. One week postfeeding, the parental strains still represented a substantial proportion of the yeast population (between 10% and 65% for BY4741, 4% and 75% for BY4742), but their presence gradually reduced over time (Fig. 1B). Conversely, the number of hybrids kept increasing over time, with the maximum observed 4 weeks after wasp feeding (Fig. 1B). At the eighth week, neither the parental strains nor the mates were found, as previously observed in hibernating wasps [14], suggesting that yeasts are not stable commensal, but transient passengers of wasps, as already observed for other insects [39], or that the use of auxotrophic strains may have limited survival in the wasp intestine. These results suggest that, in the intestine of active wasps, yeast outbreeding occurs faster than in hibernating gynes, implying that the metabolic and physical activity of wasps further promotes the process.

### Yeasts sporulate in wasp crops and germinate and ferment in wasp guts

**Figure 2:**
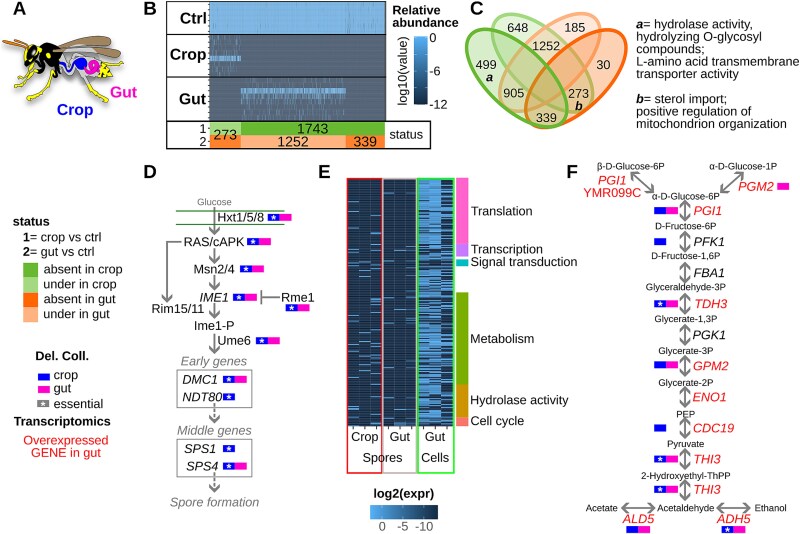
The yeast molecular response to the wasp crops and guts environment. (A) Schematic structure of the wasp intestine, with crop and gut highlighted with colors as indicated in the inset legend. (B) Heatmap of the yeast deletion strains differentially represented in the wasps’ crop and gut compared to the control samples; each row corresponds to a different sample. (C) Venn diagram comparing the list of genes necessary for the yeast survival in the wasp crop or gut with functional enrichment results (BinGO analysis, FDR < 0.05). The numbers indicate the strains not found (“absent”) or found in lower abundances (“under”) in crops or guts compared to control samples. (D) Simplified representation of the meiosis pathway (scheme gathered from KEGG) [28] and the genes found to be relevant in the wasp crop and gut throughout the deletion collection analysis. (E) Heatmap of transcriptional profiles obtained from yeast cells or spores fed to wasps and extracted from either the crop or gut after 3 days; each column corresponds to a biological replicate. The colored bar on the right of the heatmap indicates the functional activity associated with the group of genes in the corresponding rows of the heatmap (details in Table S4). (F) Simplified representation of the glycolysis pathway (scheme gathered from KEGG) [28] and the results of both deletion collection and transcriptional analysis; genes written in red are expressed by cells in the wasps’ intestine.

To investigate the association between *S. cerevisiae* and social wasps, we first examined how the wasp intestinal environment impacts yeasts. We did this by feeding wasps the *S. cerevisiae* heterozygous deletion collection—a pool of strains, each uniquely barcoded and lacking one copy of a specific gene [21]. Control samples consisted of the same pool grown in rich medium (YPD) and kept in the same room as the wasps. Four days after feeding, the relative abundance of each strain of the deletion pool was assessed through high-throughput sequencing of the wasp crop and gut contents (Fig. 2A) and compared to the corresponding relative abundance in control samples. Strains that were under-represented in intestinal samples were those lacking genes required for survival in the wasp intestine. Fewer strains were recovered from the crop (3063) compared to the control (4755) and gut (4391) (Table S1). Because the crop is the initial stage of yeast passage through the wasp intestine, the higher number of deletion strains found in the gut likely reflects rapid transit through the crop and strong selection on the yeast population remaining there. 30 min after feeding, yeast cells were observed accumulating in the proventriculus [40], with several already reaching the gut (Fig. S1). Some strains detected in the control were absent in the crop (1743) or gut (642) (Fig. 2), and 339 strains were missing from both, highlighting the genes crucial for survival in wasp intestines (Fig. 2B and C, and Table S2). Among these strains, 499 were absent in the crop but present in both the control and gut (Fig. 2C); these strains were deleted for genes enriched in hydrolase activity, hydrolysis of O-glycosyl compounds, hydrolase activity, acting on glycosyl bonds, and L-amino acid transmembrane transporter activity functions (Fig. 2B and Table S2). Conversely, 273 strains were underrepresented in the crop and were absent from the gut, suggesting stepwise selection. These strains were deleted for genes enriched in functions such as sterol import and transmembrane transport, as well as positive regulation of mitochondrial translation and organization (Table S2). This finding is in line with previous studies showing that *S. cerevisiae* is capable of synthesizing sterols, but, in anoxic conditions, it becomes a sterol auxotroph [41]. A link between sterol uptake and mitochondrial organization was ascribed in yeast to the route of sterol precursors synthesized in the inner mitochondrial membrane [42]. The deletion of only two genes, lethal in optimal conditions (the control), was tolerated in the crop or gut. The strain deleted for YJR005C-A, encoding Lso1p (a protein with a potential role in response to iron deprivation), was detected in crop samples but absent from both gut and control samples (Table S1). The strain deleted for the YDR007W gene (*TRP1*), encoding phosphoribosyl anthranilate isomerase (essential for tryptophan biosynthesis), was more abundant in the crop than in the controls or gut samples (Table S1), suggesting the presence of high levels of tryptophan in this environment. Overall, the strains identified as less represented or missing in wasp crops and guts compared to the control samples were deleted in genes related to the regulation of metabolism, molecular biosynthesis, and cell cycle and reproduction (Table S2). Focusing on the cell cycle, genes relevant in the meiotic progression were essential for survival in the wasp intestine and essential in the crop (Fig. 2D and Fig. S2), whereas genes involved in the cell cycle (vegetative) were identified in the gut (Fig. S2). This result indicates that vegetative cells have the potential to reproduce themselves and can also undergo meiosis and sporulation (and potentially mate). Most of the key regulators of the meiosis pathway (e.g. *IME1*, *UME6*, *NDT80*, *CDC14*, *SGO1*, and *REC8*) were found to be fundamental for yeast survival in the wasp crops and relevant but not necessary in the guts (Fig. 2D), and the 70% and 49% of genes previously identified as associated with the sporulation process were found to be fundamental or relevant in the crop and gut, respectively (Table S2) [43, 44]. Overall, these data indicate the potential occurrence of sporulation in the wasp crop.

To fully address molecular signatures indicating the physiological status of yeasts in the wasp intestine environment, the transcriptional profile of the SK1 strain vegetative cells and spores in the wasp crop and gut was analyzed through high-throughput sequencing analysis, using as a control the RNA extracted from wasps fed with sterile food. Whereas a large number of genes were found in cells’ transcriptional profiles (455 genes expressed in at least two samples), the transcriptional signal observed from spores was lower (135 genes expressed in at least one crop sample, 46 in at least one gut sample), but still clearly indicating spores return to vegetative settings (germination, Table S3). The transcriptional profiles were consistent among cells, but highly variable among sporal samples (Table S3). The different molecular profile observed in spores could be ascribed to asynchronous germination of spores occurring within the wasp’s intestines. Most of the genes expressed by cells in the gut, also shared with some of the spore samples from both crop and gut, were associated with translation, transcription, signal transduction, metabolism, hydrolase activity, and cell cycle (Fig. 2E and Table S4). Yeast cells’ transcripts were enriched in functions associated with the regulation of cellular metabolic processes, and in particular with the fermentative process leading to ethanol and acetate production (Fig. 2F and Fig. S2). These results are consistent with the enrichment of functions involved in sterol intake and mitochondrial organization essential for yeast survival in an anoxic/low-oxygen environment (Fig. 2C). They are also in line with recent observations of tolerance to high levels of ethanol in social wasps [45].

### Physico-chemical features of the wasp intestine expose yeast to challenging environments

**Figure 3:**
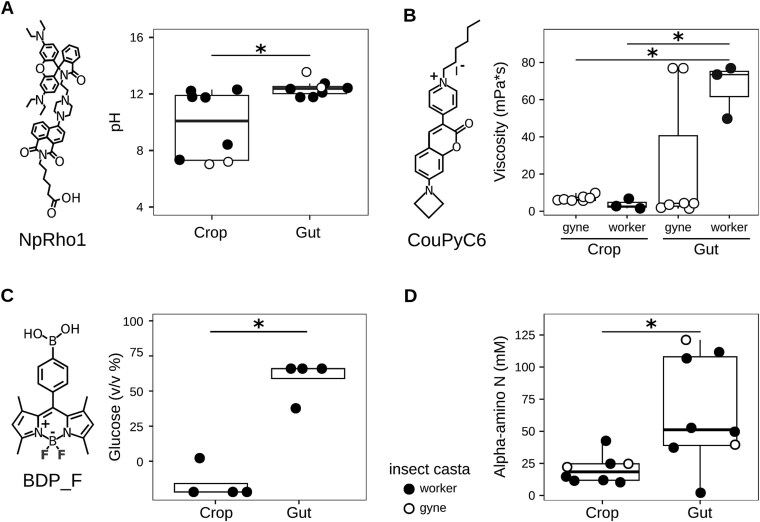
Chemico-physical characteristics of social wasp intestines. (A) Quantification of pH in the wasp crop and gut contents obtained with the fluorescent probe NpRho1; (B) quantification of gyne and worker wasp crop and gut contents’ viscosity obtained with the fluorescent probe CouPyC6; (C) quantification of glucose concentration in the wasp crop and gut contents obtained with the fluorescent probe BDP_F; (D) quantification of wasp crop and gut contents’ alpha-amino nitrogen. Calibration curves for each probe are reported in Fig. S1. ^*^ = Wilcoxon-Mann–Whitney FDR < 0.05.

The yeast molecular response to the crop and gut environments suggests that different physico-chemical properties of the two compartments impact yeast. To assess these properties, we used dedicated probes previously synthesized and validated [32, 33, 35] to measure pH, carbon sources (saccharides), nitrogen availability, and molecular-scale viscosity (Table 1). Whereas pH, saccharides, and nitrogen availability have an obvious direct impact on yeast metabolism, viscosity was included among the assessed environmental factors due to its potential impact on strain growth, e.g. through its influence on molecular transport within the intestine. After establishing a calibration curve for each probe (Fig. S4), tests were performed on both the wasp crop and gut. The wasp gut exhibited significantly more basic pH (12.4 +/− 0.6, mean +/− sd) than the crop (9.8 +/− 2.5, mean +/− sd) (Wilcoxon-Mann–Whitney *P* value: .010) (Fig. 3A, Table S5), in line with reports of intestinal pH in other insects, e.g. Argidae (Hymenoptera) and lepidopteran larvae, adult Scarabaeidae beetle, and higher termites [46]. Crop pH showed significantly higher variance (6.0) compared to gut samples (0.33, Levene test F(1,14): 12.62, *P* value: .010), suggesting a potential variation of this feature due to wasp activity (e.g. feeding or drinking). Viscosity showed significant differences when considering the intestinal compartment and the wasp casta (Fig. 3B). Indeed, whereas crop viscosity was relatively consistent among all the tested insects, worker gut viscosity was significantly higher than posthibernation founding queens (gynes) and worker crop viscosity (Wilcoxon-Mann–Whitney *P* < .05, Fig. 3B). Conversely, the viscosity of gyne guts included broader values and did not differ from worker gut or crop viscosities (Table S5). This difference may be linked to dietary and physiological traits: gynes typically consume more sugar-rich diets and store more lipids in preparation for hibernation and egg deposition, whereas workers eat more protein-rich prey [17, 47]. Regarding sugar content, the crop showed a significantly lower saccharide concentration (6 +/− 5%, mean +/− se) than the gut (67 +/− 11.7% mean +/− se) (Wilcoxon-Mann–Whitney *P* value: .023, Fig. 3C, Table S5). The glucose concentration measured in the crop content is comparable to the hexose concentration measured in *Vespa* spp. larval saliva (1.2%) [48]. The very high glucose concentration measured in the gut, not assessed in previous studies, could be ascribed to the progression of digestion in this intestinal compartment [49]. The available nitrogen differed between the two intestinal compartments, with the gut samples bearing on average a significantly higher concentration (65 +/− 43 mm) of alpha-amino nitrogen compared to the crop content (20 +/− 11 mm) (Wilcoxon-Mann–Whitney FDR = 0.021, Fig. 3D). The concentration of nitrogen measured in the crop was in line with the previous data on nitrogen consumption in *Polistes* spp. adult workers [17]. The higher concentration of sugar and nitrogen in the gut, rather than in the crop, could be ascribed to the fact that the gut is the compartment where the major food digestion occurs [17].

**Table 1:** Characteristics of chemical probes used in this study.

**Name**	**Target**	**Molecular weight**	**λ excitation (nm)**	**λ emission (nm)**	**Ref**
NpRho1	pH	863.07	one photon: 410 two photon: 760	C1 window: 480–540 C2 window: 540–600	Tan *et al.* 2024 [62]
BDP_F	Saccharides	370.01	496	508	Zhai *et al.* 2012 [32]
CouPyC6	Viscosity	490.39	488	500–600	Patawanich *et al.* 2025 [35]

**Figure 4:**
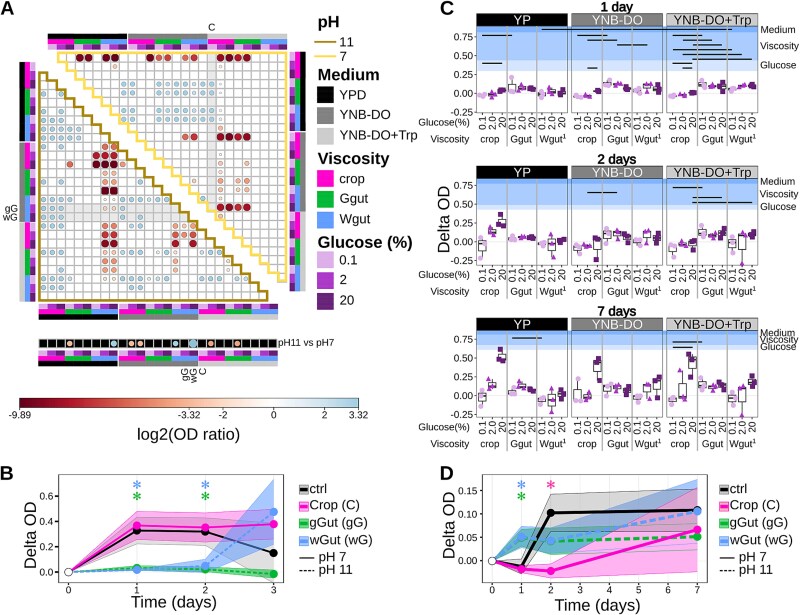
Screening of yeast cells’ survival and spores germination in conditions mimicking the wasp intestine. (A) Comparison of OD values of yeast cell cultures grown for 72 h in various settings combining various media (YP, YNB-DO, and YNB-DO+Trp), viscosities (crop = 1 mPa*s, worker gut = 37 mPa*s, and gynes gut = 52 mPa*s), glucose concentrations (0.1, 2, and 20%), and pH levels (7 - crop, 11 - gut), as indicated by the colored boxes at the four sides of the plot and listed in the legend. The points dimension and color correspond to the log2-transformed value of the ratio between the mean OD of the culture grown in the conditions indicated in the row and the mean OD of the culture grown in the conditions indicated in the column; only data significantly differing between the compared conditions are shown (Wilcoxon-Mann–Whitney FDR < 0.05). Black-background boxes report the comparison between cultures grown in the same conditions but at different pH (the OD at pH 11 divided by the OD at pH 7). Gray background boxes highlight the condition indicated by the number on the top of the column, or on the left of the row, corresponding to the wasp crop environment (C), the worker wasp gut (gG), or the gyne wasp gut (wG). (B) Comparison of OD measured at different time points for cells grown under the conditions corresponding to wasp crop, gyne gut, worker gut, and control (YP, 2% glucose, 0.9 mPa*s); the asterisks show significant differences between the control and the condition indicated by the color (Wilcoxon-Mann–Whitney FDR < 0.05). (C) Comparison of spore germination. The delta OD (obtained as the difference between the culture OD measured at the indicated time point and the OD of the same culture measured at inoculation) is reported for the three biological replicates performed for all the tested conditions, described as in panel a. (D) Comparison of OD measured at different time points for spores grown under the conditions corresponding to wasp crop, gyne gut, worker gut, and control (YP, 2% glucose, 0.9 mPa*s); the asterisks indicate significant differences between the control and the condition indicated by the color (Wilcoxon-Mann–Whitney FDR < 0.05).

### Crop and gut environmental features allow yeast cell growth and sporal germination

Having defined the described physico-chemical characteristics of the wasp intestine, we could then evaluate in a controlled system whether the environment per se can promote the yeast mating process. To this end, the growth potential of vegetative cells and spores was monitored in media simulating the conditions observed in wasp crops and guts (in static cultures, mimicking semi-anoxic conditions). Intestinal environments were simulated with a minimal medium (YNB-DO, Yeast Nitrogen Base with drop-out) and specific features characterizing the crop and gut. For crop-mimicking conditions, YNB-DO was supplemented with 50 mg/l tryptophan (YNB-DO+Trp). The crop was simulated with 1 mPa*s viscosity, 2% glucose, and pH 7; the gyne gut with 37 mPa*s viscosity, 20% glucose, and pH 11; the worker gut with 52 mPa*s viscosity, 20% glucose, and pH 11. Despite the glucose concentration measured in the gut being higher (67%) than the one used to mimic this environment, we decided to perform the simulation using the concentration i.e. commonly found in mature wine grapes, one of the wasps’ natural foods with the highest sugar concentration [9] to allow the precise adjustment of the other tested factors (e.g. viscosity). The optimized medium for *S. cerevisiae* growth (YP) was used as a control, supplemented with 2% glucose. The growth of yeast cells and germination of yeast spores were evaluated across all the possible combinations of medium, viscosity, glucose, and pH (Fig. 2), focusing on crop and gut-specific settings. Yeast cells were able to grow in most of the tested settings, except for conditions combining gut pH (pH 11) with crop and gyne viscosities, or crop pH (pH 7) combined with gyne and worker gut viscosities (Fig. S5). Under basic pH condition, cell reproduction was strongly associated with medium viscosity: cells inoculated in less viscous (1 mPa*s) YP medium grew significantly less than cells inoculated in more viscous YP (37 mPa*s and 52 mPa*s, gut viscosities), YNB-DO and YNB-DO+Trp media (Wilcoxon-Mann–Whitney test FDR < 0.05, Fig. 4A). The crop-like environment (YNB-DO+Trp, 1 mPa*s viscosity, pH 7, 2% glucose), allowed substantial cell growth, comparable to the optimized laboratory conditions (Wilxocon-Mann–Whitney FDR > 0.05, Fig. 4B). Comparison of growth under different pH conditions revealed that the basic environment was significantly better tolerated than the neutral one, but only in the absence of tryptophan (YNB-DO), with high glucose concentration, and gut-like viscosity conditions (boxes in dark background, Fig. 4A). This corresponds to the environmental characteristics of worker wasp guts (Fig. 3). It is also worth noting that the worker gut conditions promoted the growth of cells only on the third day postinoculation, possibly indicating a longer period of adaptation required by the cells in this environment (Fig. 4B). In addition, the viability and numerosity in the media simulating the crop, the gut, and the control conditions, showed no significant differences among the tested environments (Wilcoxon-Mann–Whitney FDR > 0.05), but cultures grown in gut-like conditions formed large cell clusters, previously recognized as a yeast protective response to cellular stresses [50] and favouring the inter-strain mating [51] (Fig. S6). In addition, spores exposed to environments associated with high viscosity (observed in worker wasp guts) were not able to germinate in the rich nutrient medium (YPD), but they germinated in the minimal medium (YNB), supplemented or not with tryptophan and with high glucose concentration (20%) (Fig. S7). In YNB-DO+Trp, the spore germination was significantly higher in the high-viscosity condition (53 mPa*s, as in the worker wasp gut) than in the same medium with lower viscosity or in the YP medium with high viscosity (Wilcoxon-Mann–Whitney test FDR < 0.05, Fig. 4C). This specific combination of physico-chemical characteristics, mimicking the worker wasp gut, favors rapid spore germination. Finally, spores inoculated in the media simulating gyne and worker guts’ conditions showed a significantly higher OD compared to the control settings (spores in YPD) after one day of treatment (Wilcoxon-Mann–Whitney FDR < 0.05, Fig. 4D).

Overall, our in vitro analyses show that yeast cells can survive and grow both in the crop and in the gut, albeit with a short delay in the latter. Conversely, yeast spore germination is faster in the gut than in the crop.

### Computational simulations confirm the yeast metabolism adaptation in the wasp intestine environment

**Figure 5:**
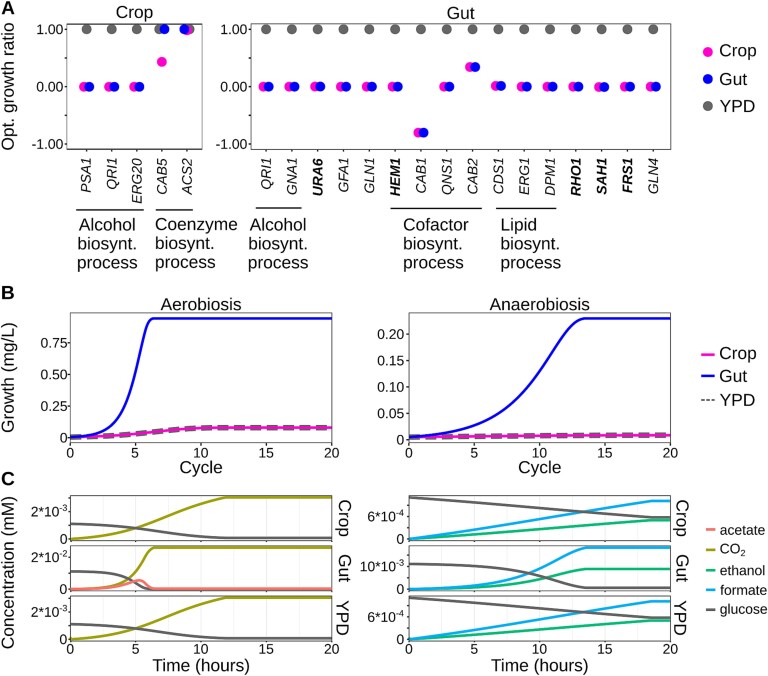
Computational simulation analysis of the impact of crop and gut environment on yeast growth. (A) Genes whose elimination from the computational simulation results in no growth or lower growth compared to the optimal conditions, YPD. opt. The ratio of the predicted growth in the tested conditions divided by the predicted growth in the optimal conditions is reported. At the bottom of each plot, the gene ontology molecular function of the grouped genes is reported. (B and C) Growth curve (B) and metabolites consumption and production (C) in crop, gut, and optimal conditions.

The definition of yeast molecular and physiological responses to the social wasp intestinal environment and the physico-chemical characterization of the crop and the gut provided fundamental information suitable for further investigation at the metabolic level. A computational simulation (Fig. 5A and Table S6) was performed to assess the impact on yeast growth potential of the deletion of essential genes. With this aim, a model simulating the crop and gut environmental conditions (Fig. 4) was run by excluding one at a time the genes found to be essential or necessary in the deletion collection analysis (Fig. 2A), and the results were compared with the results of the model in the same conditions and with all the genes included. The analysis highlighted the essential role of genes involved in the alcohol biosynthesis process in both the wasp crop (*PSA1*, *QRI1*, and *ERG20*) and gut (*QRI1* and *GNA1*). This set of genes was also expressed in yeast cells (Fig. 2E). In addition, genes involved in coenzyme (*CAB5* and *ACS2*) and cofactor (*HEM1*, *CAB1*, *QNS1*, and *CAB2*) biosynthetic processes were essential in crop and gut, respectively (Fig. 5A), suggesting that yeasts in the wasp intestinal environment need to produce cofactors, a fundamental resource for the host. The observation of essential genes involved in lipid biosynthetic processes (*CDS1*, *ERG1*, and *DPM1*) could be an indication of yeast tolerance to ethanol, as previously reported [52]. A second computational simulation was performed with the COMETS platform [36] to predict the growth and metabolite utilization/production in conditions mimicking the crop and gut. This revealed a greater growth potential of yeasts in gut conditions compared to both crop and optimal (YPD) conditions for both aerobic and anoxic settings (Fig. 5B). The model highlighted that, in aerobic conditions, yeasts produce CO_2_ in both crop and gut settings, with the addition of acetate production in the gut (Fig. 5C). In anoxic conditions, the most relevant yeast metabolites are formic acid and ethanol (Fig. 5C). The latter parallels the yeast transcriptional profile observed in the wasp gut (Fig. 2E), and the relevance of alcohol biosynthesis genes observed through deletion collection analysis and computational model (Fig. 2A and 5A). Tolerance to formic acid was observed as a peculiar characteristic of yeasts isolated from insects [53], conferring an advantage to the host by contributing to control fungal pathogens [54].

## Conclusions

Dissecting the chemico-physical properties of *Polistes* wasps’ intestines and analysing *S. cerevisiae* molecular and physiological responses provides fundamental insights into the yeast-insect association and yeast ecology. As yeasts pass through the wasp intestine, they encounter two distinct environments. The crop shows conditions similar to YPD, the optimized medium for *S. cerevisiae* growth, but with a slightly higher viscosity and tryptophan concentration. This environment is highly dynamic due to wasp activities: abdominal tracheal extension can compress the crop rapidly [55]; worker wasps contract the crop to feed larvae and nest mates via trophallaxis [56]; in spring, gynes and workers abrade wood to build nests [57]. Our molecular and physiological analyses show that in the crop, yeast can survive and reproduce while also being exposed to an environment demanding sporulation. In contrast, the gut presents harsher conditions -basic pH and high glucose concentration and viscosity- but offers abundant nutrients (nitrogen and glucose). These settings support slower but sustained growth via alcoholic fermentation, with production of ethanol and formic acid. The pH and glucose concentrations measured in the gut content are higher than expected. Although gut pH shows variability across insect orders and families, alkaline midguts have been documented in hymenopteran larvae of the family Argidae, lepidopteran caterpillars, xylophagous beetles, and higher termites [45]. These patterns are often linked to diet, with basic pH favouring the activity of proteases (e.g. trypsin and aminopeptidases) and acidic values preserving the activity of amylases and maltases [46]. In *Polistes* spp., comparisons are less straightforward, given their generalist diet combining sugary resources with insect prey [57]. The very high glucose concentration we observed in the gut of social wasps has, to our knowledge, never been reported previously. By contrast, the values measured in the crop fall within the range of hexose concentrations found in larval saliva of other social wasps (hornets, *Vespa* spp.) [46, 58]. The previous data on social wasp larval secretions revealed high levels of amino acids [48], hence supporting our hypothesis that *S. cerevisiae* cells lacking key genes for tryptophan biosynthesis were still able to survive in the wasp intestine, presumably benefiting from host-derived amino acids.

Spore germination occurs faster in the gut than in the crop and the optimal YPD conditions. Observed cell aggregation in the gut may enhance mating by promoting close contact between cells. Overall, our findings suggest that diploid yeast cells sporulate in the crop and, once transferred to the gut, spores germinate and potentially mate. The transcriptional analysis performed in our study, based on the response of yeasts three days after wasp feeding, provides a first hint of the molecular response of yeasts to the wasp intestinal environment, but it’s only a partial view of the complete response triggered by this association and should be further investigated. Further research is needed to understand whether ascus rupture, necessary for yeast outbreeding, occurs in the gut, possibly triggered by mechanical stress (e.g. compression of the crop or passage through the proventriculus) [59] or host-derived enzymes.

Our findings deepen the understanding of the strict specificity of the *S. cerevisiae*-social wasps interactions. Social wasps may tolerate the ethanol produced by yeasts in their guts [45], and benefit from yeast-derived formic acid, which has antimicrobial effects against fungal pathogens [54]. Characterizing the social wasp intestine environment, combining sequential environments exposing yeasts to a series of events promoting their outbreeding, provides a foundation for exploring a potentially mutualistic relationship influencing the evolution of both partners. For yeasts, outbreeding helps preserve genetic diversity by maintaining mutations in heterozygosity [60]. For wasps, the strict association with an ethanol-producing microorganism may have supported the development of eusociality by limiting food competition (competitors do not tolerate ethanol) and by supplying pathogen-free nourishment to offspring and nestmates [61].

## Supplementary Material

Supplementary_materials_last_wraf243

## Data Availability

The yeast deletion collection sequencing datasets generated and analyzed during the current study are available in the NCBI Sequence Read Archive (https://www.ncbi.nlm.nih.gov/sra), under BioProject ID PRJNA1267698 (BioSample accessions: SAMN48727422-SAMN48727443). Transcriptional (RNAseq) raw data generated and analyzed during the current study are available in the NCBI Sequence Read Archive (https://www.ncbi.nlm.nih.gov/sra), under BioProject ID PRJNA1267698 (BioSample accessions: SAMN48727410-SAMN48727421). The scripts used to perform the Dynamic flux balance analysis are available in GitHub (https://github.com/segrelab/waspgut).
