# Supplementary material for: Wasp intestinal cues drive yeast toward outbreeding strategies
Source: ISME J. 2025 Nov 1;19(1):wraf243. doi: 10.1093/ismejo/wraf243 (PMC12642876; doi:10.1093/ismejo/wraf243)

Supplementary material for

Wasp intestinal cues drive yeast toward outbreeding strategies

Silvia Abbà[1], Liam D. Adair[4], Francesca Barbero[1], Luca P. Casacci[1], Iljia Dukovski[2], Francisca Font[3], Tom Hawtrey[4], Elizabeth J. New[4], Jukkrit Nootem[4], Pramsak Patawanich[4], Lukas Patten[2], Marco Polin[3], Daniel Segrè [2], Nian Kee Tan[4], Irene Stefanini[1]

[1] Department of Life Sciences and Systems Biology, University of Turin, Turin, Italy
[2] Bioinformatics Program, Faculty of Computing and Data Sciences, Boston University, Boston, MA, USA.
[3] Mediterranean Institute for Advanced Studies, IMEDEA UIB-CSIC, C/Miquel Marqués 21, 07190, Esporles, Spain.
[4] School of Chemistry, The University of Sydney, NSW 2006, Australia; Australian Research Council Centre of Excellence for Innovations in Peptide and Protein Science, The University of Sydney, NSW 2006, Australia

This pdf includes:

- Supplementary details

1. Technical details on Yeast deletion collection analysis
2. Technical details on transcriptomics analysis
3. Calibration analyses for fluorescent probes
    3.1 Calibration analyses for fluorescent probes testing pH
    3.2 Calibration analyses for fluorescent probes testing glucose concentration
    3.3 Calibration analyses for fluorescent probes testing viscosity
    3.4 Calibration analyses for alpha-amino nitrogen quantification

- Supplementary Figures

Fig. S1: Reconstruction of a social wasp intestine and focus on the distribution of *Saccharomyces cerevisiae* cells.
Fig. S2: Annotation of yeast deletion collection results on the cell cycle (a) and meiosis (b) KEGG pathways.
Fig. S3: Annotation of yeast deletion collection and transcriptomics results on the glycolysis/gluconeogenesis KEGG pathway.
Fig. S4: Calibration curves for fluorescent probes used for the evaluation of pH, saccharides, and viscosity values in social wasp intestines.
Fig. S5: Growth of yeast cells in environmental conditions mimicking the wasp intestine.
Fig. S6: Evaluation of yeast cell viability in the crop and gut environment.
Fig. S7: Germination and growth of yeast spores in environmental conditions mimicking the wasp intestine.

Additional supplementary materials (provided as independent files):

Table S1: Results of yeast heterozygous deletion collection treatments.
Table S2: Yeast heterozygous deletion collection treatments - Gene Ontology enrichment results.
Table S3: Results of NGS transcriptomics analysis of yeast cells and spores in social wasp intestines.

Table S4: Functional enrichment of genes found in NGS transcriptomics analysis of yeast cells and spores in social wasp intestines.

Table S5: Saccharides and available nitrogen concentrations, viscosity, and pH values measured in wasp intestines.

Table S6: Results of computational simulations.

Growth_curve.ipynb: Script used to evaluate the growth curve and metabolites consumption and production with COMETS. The sections starting with "minimal medium aerobic", "minimal medium with anaerobiosis", "YPD with aerobiosis", "YPD with anaerobiosis", "gut with aerobiosis", "gut with anaerobiosis", "crop with aerobiosis", and "crop with anaerobiosis" were run alternatively to perform the analysis on the different tested media/conditions. The code is available on GitHub, https://github.com/segrelab/waspgut

GENETICS_deletion.ipynb: script used to perform the gene deletion analysis in COMETS. The sections starting with "MIMNAL YEAST MEDIUM", "YPD", "crop", and "gut" were run alternatively to perform the analysis on the different tested media/conditions. The code is available on GitHub, https://github.com/segrelab/waspgut

Supplementary details

1. Technical details on Yeast deletion collection analysis

The Yeast Deletion Collection is made of 5082 diploid strains, each deleted in gene and recognizable through a specific sequence tag (20 bp unique tag sequences assigned to each ORF). Before the experiment, the yeast deletion collection pool was grown for 2 hours in YPD (1% yeast extract, 2% peptone, 2% glucose).

The amount and quality of the extracted DNA were assessed with NanoDrop spectrophotometer. To identify and quantify the deletion strains present in the intestinal content, the genomic region including the strain-specific tag was amplified using the primers UpTagDel (5′-GATGTCCACGAGGTCTCT-3′) and Kan_in_RV (5′-CTGCAGCGAGGAGCCGTAAT-3′), as described in Giaever and Nislow. For the sequencing library preparation, the primers were extended with sequences suitable for Illumina adaptors addition, resulting in the primer FW: 5'-ACACTCTTTCCCTACACGACGCTCTTCCGATCTGATGTCCACGAGGTCTCT-3' and RV: 5'-GACTGGAGTTCAGACGTGTGCTCTTCCGATCTCTGCAGCGAGGAGCCGTAAT-3'.

PCR amplification was performed with Taq-Platinum HiFi Polymerase (Thermo Fisher) and the following thermal cycle: 94°C for 2 minutes, (94°C for 30 seconds, 60°C for 30 seconds, 72°C for 30 seconds) for 30 cycles, final extension 72°C for 2 minutes. The resulting amplified DNA fragments, upon checking on horizontal gel electrophoresis, were purified using the E.Z.N.A.® Cycle Pure Kit (Omega Bio-Tek).

2. Technical details on transcriptomics analysis

SK1 strain cells were obtained by an overnight culture in YPD medium, at 28°C with shaking. Yeast tetrads were obtained after growing the SK1 strain in YPD for 48 hours, washing the cell pellet twice with sterile water, resuspending the pellet in 2% potassium acetate, and incubating at 28°C for at least 3 days, until the sporulation rate was higher than 80%. To eliminate any potential vegetative cells, tetrads were treated as previously described with slight modifications. Briefly, tetrads were washed twice with sterile water, extracted with diethyl ether (vol 1:1), and mixed for 2 hours at RT. The upper phase (ether) was then removed, and this step was repeated twice. The remaining pellet was resuspended in sterile water to get the proper tetrad concentration to feed wasps.

3. Calibration analyses for fluorescent probes

3.1 Calibration analyses for fluorescent probes testing pH

The analysis for the evaluation of the pH of social wasps' intestinal content was carried out with the probe NpRho1.

The calibration curve for the probe NpRho1 (20 µM), suitable for pH quantification, was obtained by incubating NpRho1 (20 µM) at R.T. for 30 minutes with 10 µL of three standard solutions (pH 4.0, 7.0, and 13.0) in triplicate. Deionized water was used as a blank. Fluorescence was measured at 580 nm and 530 nm after excitation at 410 nm. The ratio of the fluorescence intensity at 580 nm divided by the fluorescence intensity at 530 nm was calculated and fitted with the corresponding pH value using the lm R function.

Discrepancies in the fluorescence intensity were observed among different measurements of reference samples; hence, a new calibration curve was obtained for each tested group of samples (different colors in **Supplementary Figure 4**). Linear and polynomial tests were

performed to identify the model best fitting (the model with the highest R^2 value) with the calibration data; the resulting values are reported in the following table for comparison.

<table>
<tr><th></th><th></th><th></th><th>Estimate</th><th>Std. Error</th><th>t-value</th><th>Pr(>|t|)</th><th>Residual standard error</th><th>Adjusted R^2</th></tr>
<tr><td rowspan="5">green</td><td rowspan="2">fluoRatio~e+ a(pH)</td><td>(Intercept)</td><td>0.779</td><td>0.029</td><td>26.07</td><td>1.3E-12</td><td rowspan="2">0.047 on 13 degrees of freedom</td><td rowspan="2">0.80</td></tr>
<tr><td>pH</td><td>-0.025</td><td>0.003</td><td>-7.57</td><td>4E-6</td></tr>
<tr><td rowspan="3">fluoRatio~e+ a(pH)+ b(pH)^2</td><td>(Intercept)</td><td>0.953</td><td>0.076</td><td>12.52</td><td>3E-8</td><td rowspan="3">0.0.38 on 12 degrees of freedom</td><td rowspan="3">0.87</td></tr>
<tr><td>pH</td><td>-0.082</td><td>0.021</td><td>-3.98</td><td>0.002</td></tr>
<tr><td>pH^2</td><td>0.003</td><td>0.001</td><td>2.81</td><td>0.016</td></tr>
<tr><td rowspan="5">red</td><td rowspan="2">fluoRatio~e+ a(pH)</td><td>(Intercept)</td><td>1.146</td><td>0.044</td><td>25.90</td><td>2E-10</td><td rowspan="2">0.065 on 10 degrees of freedom</td><td rowspan="2">0.90</td></tr>
<tr><td>pH</td><td>-0.051</td><td>0.005</td><td>-10.22</td><td>1E-6</td></tr>
<tr><td rowspan="3">fluoRatio~e+ a(pH)+ b(pH)^2</td><td>(Intercept)</td><td>1.582</td><td>0.010</td><td>153.01</td><td><2E-16</td><td rowspan="3">0.005 on 9 degrees of freedom</td><td rowspan="3">0.9995</td></tr>
<tr><td>pH</td><td>-0.175</td><td>0.003</td><td>-62.11</td><td>4E-13</td></tr>
<tr><td>pH^2</td><td>0.007</td><td>0.001</td><td>44.28</td><td>8E-12</td></tr>
<tr><td rowspan="5">blue</td><td rowspan="2">fluoRatio~e+ a(pH)</td><td>(Intercept)</td><td>1.716</td><td>0.140</td><td>12.25</td><td>2E-8</td><td rowspan="2">0.23 on 13 degrees of freedom</td><td rowspan="2">0.72</td></tr>
<tr><td>pH</td><td>-0.096</td><td>0.016</td><td>-6.04</td><td>4E-5</td></tr>
<tr><td rowspan="3">fluoRatio~e+ a(pH)+ b(pH)^2</td><td>(Intercept)</td><td>3.292</td><td>0.019</td><td>169.76</td><td><2E-16</td><td rowspan="3">0.009 on 12 degrees of freedom</td><td rowspan="3">0.9995</td></tr>
<tr><td>pH</td><td>-0.543</td><td>0.005</td><td>-102.80</td><td><2E-16</td></tr>
<tr><td>pH^2</td><td>0.026</td><td>0.001</td><td>85.36</td><td><2E-16</td></tr>
</table>

3.2 Calibration analyses for fluorescent probes testing glucose concentration

The analysis for the evaluation of the saccharide concentration in social wasps' intestinal content was carried out with the probe BDP_F. Probe stability was compared among multiple reference samples, resulting in stable fluorescence emission; hence, a reference set of calibrating samples was used to obtain a polynomial calibration curve that was used to convert probe fluorescence in samples into saccharide concentration. Every sampled test was performed by including calibration samples to confirm the consistency of measurements. The calibration curve for the probe BDP-F (10 μM), suitable for saccharide measurements, was obtained by using 10 μL of standard glucose solutions (700.0 g/L, 500.0 g/L, 250.0 g/L, 100.0 g/L, and 20.0 g/L). Each experiment was conducted in quadruplicate to ensure reliability and reproducibility; the measured fluorescence (excitation 496 nm, emission 508 nm) was then fitted against the corresponding glucose concentration.
Linear and polynomial tests were performed to identify the model best fitting (the model with the highest R^2 value) with the calibration data; the resulting values are reported in the following table for comparison.

<table>
<tr><th></th><th></th><th>Estimate</th><th>Std. Error</th><th>t-value</th><th>Pr(>|t|)</th><th>Residual standard error</th><th>Adjusted R^2</th></tr>
<tr><td rowspan="2">fluorescence~e+ a(glucose)</td><td>(Intercept)</td><td>176.42</td><td>11.808</td><td>14.94</td><td>1.2E-12</td><td rowspan="2">41.19 on 21 degrees of freedom</td><td rowspan="2">0.96</td></tr>
<tr><td>glucose</td><td>7.32</td><td>0.327</td><td>22.4</td><td>3.9E-16</td></tr>
<tr><td rowspan="3">fluorescence~e+ a(glucose)+ b(glucose)^2</td><td>(Intercept)</td><td>197.03</td><td>10.24</td><td>19.24</td><td>2.3E-14</td><td rowspan="3">31.12 on 20 degrees of freedom</td><td rowspan="3">0.98</td></tr>
<tr><td>glucose</td><td>3.53</td><td>0.96</td><td>3.68</td><td>0.001</td></tr>
<tr><td>glucose^2</td><td>0.06</td><td>0.01</td><td>4.10</td><td>0.001</td></tr>
</table>

3.3 Calibration analyses for fluorescent probes testing viscosity

The analysis for the evaluation of the viscosity of social wasps' intestinal content was carried out with the probe CouPyC6. Viscosity data for PEG 4000 as a function of concentration (expressed as grams of solute per cm^3 of solution) at 298.15 K were obtained from Kirincic and Klofutar (1999), as reported in the table below.

PEG 4000 (g/cm^3)(g/ml)	Viscosity (mPL)
0.061	0.968
0.0121	1.050
0.0170	1.119
0.0243	1.229
0.0340	1.388

0.0486	1.660
0.0680	2.076
0.0777	2.321
0.0971	2.866

Table: data reporting the relation between viscosity (mPL) and concentration of PEG 4000 (g/ml) (Kirincic and Klofutar, 1999).

These values were used to obtain a polynomial function suitable for expanding the viscosity range and including values potentially present in wasp intestines (see the figure below). Then, the calibration curve relating the medium viscosity (calculated with the previously described formula from PEG 4000 concentrations) to the fluorescence emitted by the CouPyC6 (0.5 μM) was obtained using polyethylene glycol 4000 solutions (PEG 4000, Sigma-Aldrich) in deionized water at concentrations of 500.0 g/L, 350.0 g/L, 250.0 g/L, 125.0 g/L, 62.5 g/L, and 0.0 g/L (**Supplementary Figure 4**). Glucose solutions at several concentrations (800.0 g/L, 500.0 g/L, 220.0 g/L, 70.0 g/L, 20.0 g/L) were prepared to assess the influence of glucose on viscosity. The probe CouPyC6 was added to each solution to a final concentration of 0.5 μM. Fluorescence measurements (excitation 488 nm, emission 500-600 nm) were conducted in five replicates for each condition. The fluorescence intensity was then fitted against the viscosity value of the corresponding sample by using the lm R function. Discrepancies in the fluorescence intensity were observed among different measurements of reference samples; hence, every test was performed with the inclusion of reference samples (PEG 4000 at the concentrations used for the generation of the calibration curve), and a scaling value, calculated as the ratio between fluorescence emissions of the same sample in different sets, was used to normalize the measurements (indicated in the "Calibrated_Florescence" column of **Supplementary Table 5**). To reduce the impact of error propagation occurring, rather than evaluating the viscosity of wasp intestine samples diluted with water and evaluating the relation between diluted viscous samples (e.g., dilution PEG 4000 concentration), expected viscosity, and probe fluorescence, we preferred to proceed with the definition of the confidence interval for the relationship between fluorescence and viscosity. The range of viscosity values that could be measured according to the described approach was evaluated by determining the local minimum and maximum values of the polynomial calibration curve relating the viscosity to the fluorescence intensity (details in **Supplementary Figure 4c**). Wasp intestine samples showing fluorescence intensity lower than this range were considered to have a viscosity similar to that of water (0.8991 mPL); wasp intestine samples showing fluorescence intensity higher than the confidence interval were considered to have a viscosity higher than the maximal measurable viscosity value (76.756 mPL). Linear and polynomial tests were

performed to identify the model best fitting (the model with the highest R^2 value) with the calibration data; the resulting values are reported in the following table for comparison.

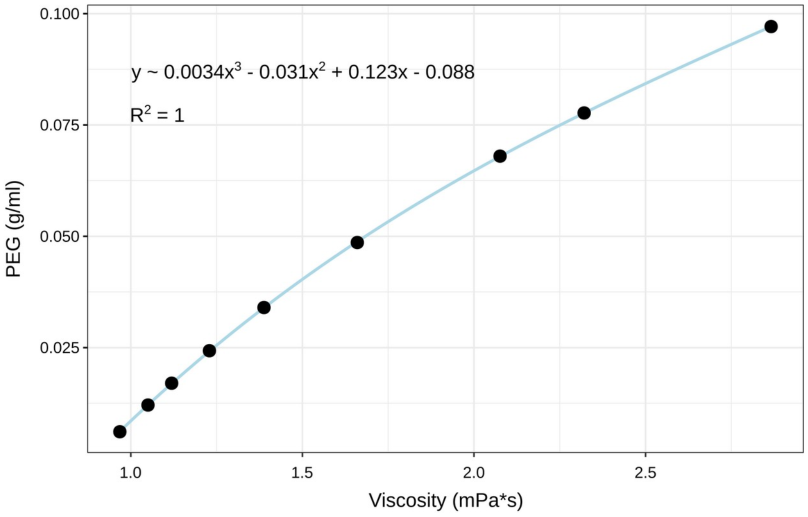

		**Estimate**	**Std. Error**	**t-value**	**Pr(>|t|)**	**Residual standard error**	**Adjusted R^2**
**fluorescence~e+ a(viscosity)**	(Intercept)	-0.036	0.004	-10.02	2E-5	0.004 on 7 degrees of freedom	0.99
	Viscosity	0.048	0.002	23.40	7E-8		
**fluorescence~e+ a(viscosity)+ b(viscosity)^2**	(Intercept)	-0.068	0.002	-29.71	1E-7	0.001 on 6 degrees of freedom	0.99
	Viscosity	0.087	0.002	32.16	6E-8		
	Viscosity^2	-0.010	0.001	-14.45	7E-6		
**fluorescence~e+ a(viscosity)+ b(viscosity)^2+ c(viscosity)^3**	(Intercept)	-0.088	0.002	-55.08	4E-8	0.001 on 5 degrees of freedom	1
	Viscosity	0.1232	0.003	43.78	1E-7		
	Viscosity^2	-0.031	0.002	-19.72	6E-6		
	Viscosity^3	0.004	0.001	13.05	4E-5		

3.4 Calibration analyses for alpha-amino nitrogen quantification

The dedicated Steroglass kit for alpha-amino nitrogen concentration was used to evaluate the concentration of alpha-amino nitrogen in the wasp crop and gut. Various concentrations of leucine (2 mM, 4 mM, 6 mM, 8 mM) were used to obtain a calibration curve (**Supplementary Figure 4d**). Crop and gut contents were diluted in water to ensure the amount of measured nitrogen was within the limits of the calibration curve. Linear and polynomial tests were performed to identify the model best fitting (the model with the highest R^2 value) with the calibration data; the resulting values are reported in the following table for comparison.

<table>
<tr><th></th><th></th><th>Estimate</th><th>Std. Error</th><th>t-value</th><th>Pr(>|t|)</th><th>Residual standard error</th><th>Adjusted R^2</th></tr>
<tr><td rowspan="2">DeltaOD~e+ a(Alpha-amino nitrogen)</td><td>(Intercept)</td><td>0.093</td><td>0.004</td><td>22.59</td><td>4E-14</td><td rowspan="2">0.010 on 17 degrees of freedom</td><td rowspan="2">0.997</td></tr>
<tr><td>Alpha-amino nitrogen</td><td>0.051</td><td>0.001</td><td>62.10</td><td>< 2E-16</td></tr>
<tr><td rowspan="3">DeltaOD~e+ a(Alpha-amino nitrogen)+b(Alpha-amino nitrogen)^2</td><td>(Intercept)</td><td>0.086</td><td>0.005</td><td>18.66</td><td>3E-12</td><td rowspan="3">0.008 on 16 degrees of freedom</td><td rowspan="3">0.996</td></tr>
<tr><td>Alpha-amino nitrogen</td><td>0.057</td><td>0.002</td><td>201.93</td><td>2E-13</td></tr>
<tr><td>Alpha-amino nitrogen^2</td><td>-0.001</td><td>0.001</td><td>-2.40</td><td>0.029</td></tr>
</table>

Supplementary Figures

Fig. S1

Reconstruction of a social wasp intestine and focus on the distribution of *Saccharomyces cerevisiae* cells. Cells of the SK1 *S. cerevisiae* strain, after overnight incubation in YPD, were stained with Rose Bengal and fed to social wasps. Thirty minutes after the feeding, wasps were killed, and their intestines were observed with fluorescence microscopy. The image of the intestine is the reconstruction of sequential pictures taken with 2X magnification; focus on different sections of the intestine was made with 40X magnification to observe yeast cells (some examples of yeast cells are highlighted with white arrows).

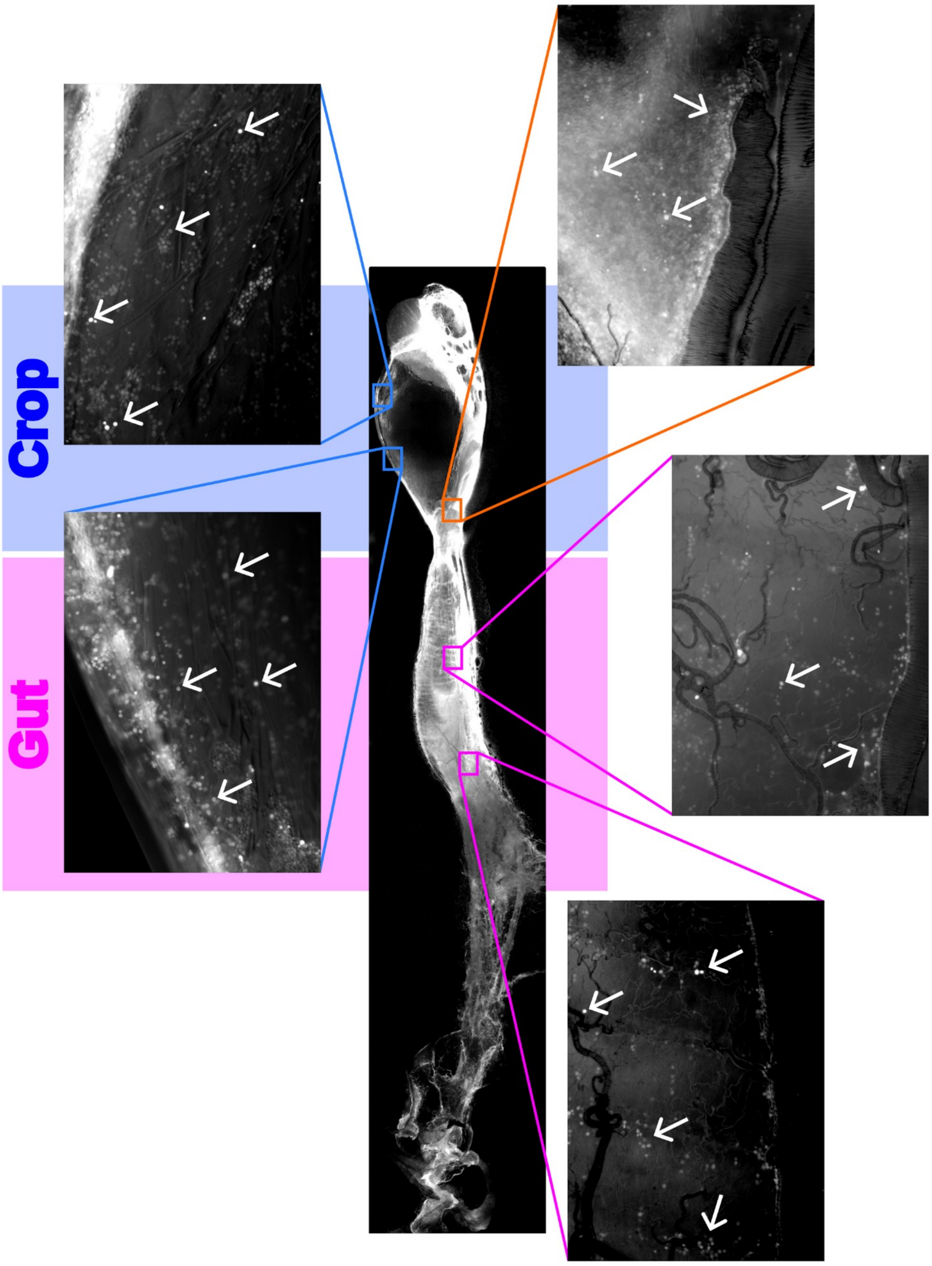

Fig. S2

Annotation of yeast deletion collection results on the cell cycle (a) and meiosis (b) KEGG pathways. Schematic diagrams were obtained from KEGG database.

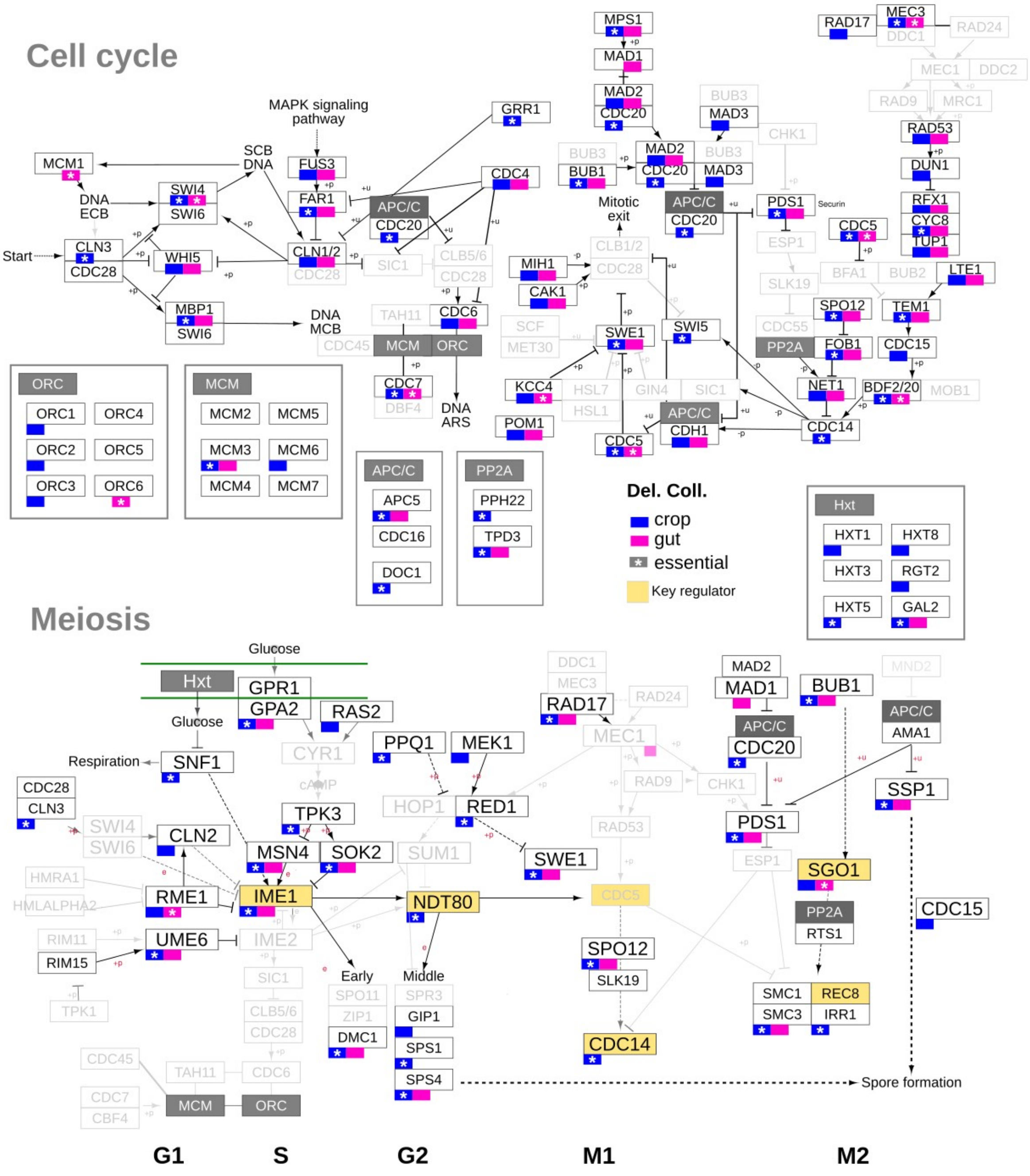

Fig. S3

Annotation of yeast deletion collection and transcriptomics results on the glycolysis/gluconeogenesis KEGG pathway. Schematic diagrams were obtained from KEGG database.

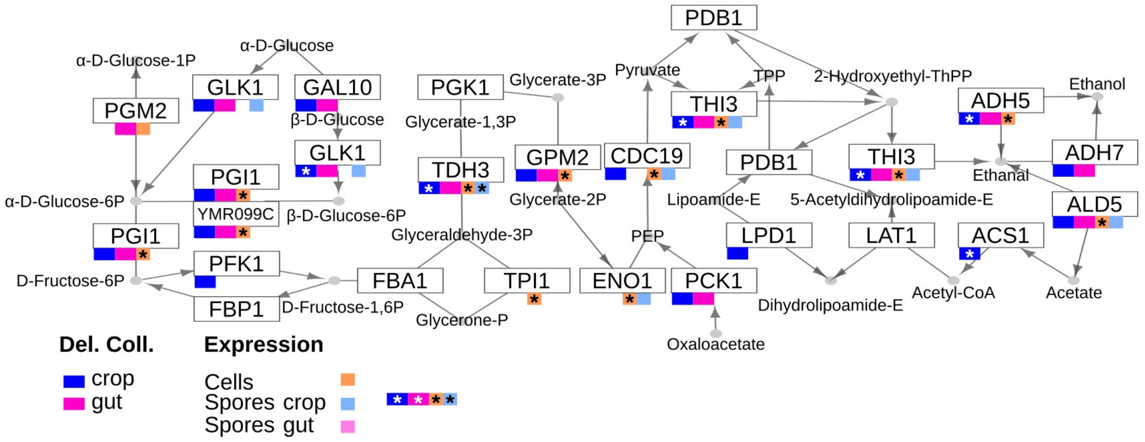

Fig. S4

Calibration curves for fluorescent probes used for the evaluation of pH, saccharides, viscosity, and alpha-amino nitrogen in social wasp intestines. a) The calibration curve for pH measurement was performed using the probe NpRho1 and standard pH solutions (details in materials and methods). **b)** The calibration curve for saccharide concentration evaluation was performed with the BDP_F probes and glucose as standard (details in materials and methods). **c)** The calibration curve for viscosity evaluation was performed with the CouPyC6 and various concentrations of PEG (details in materials and methods). Further details on the analytic process are reported in the supplementary details section. **d)** The calibration curve for alpha-amino nitrogen concentration was performed with the dedicated kits (Steroglass), as described in the detailed protocol. **a-d)** The regression functions, used to convert the measured value in samples to the corresponding factor (pH, sucrose concentration, viscosity, or nitrogen), were obtained with the lm R function; the details are reported at the beginning of this document. Raw data of calibration samples and wasp samples are reported in **Supplementary Table 5**.

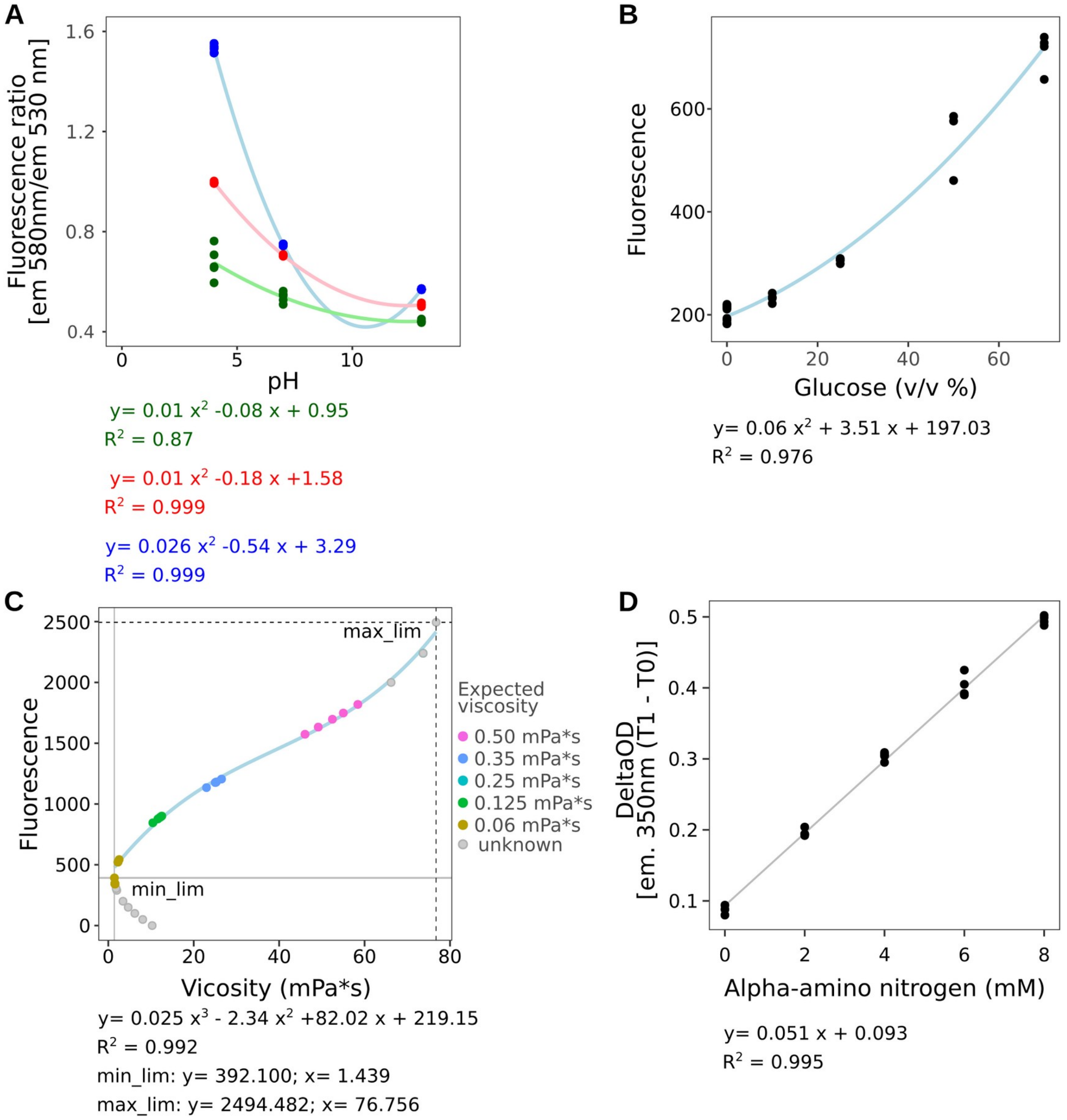

Fig. S5

Growth of yeast cells in environmental conditions mimicking the wasp intestine. The plots indicate the average delta OD (obtained as the difference between the culture OD measured at the indicated time point and the OD of the same culture measured at the moment of inoculation) and standard deviation (error bars) of three biological replicates. Medium, pH, and viscosity are indicated on top of each plot; glucose is indicated as shown in the color legend. Asterisks indicate conditions resulting in a delta OD significantly different from the one measured at the inoculation time, with the color corresponding to the concentration of glucose; ashtags indicate conditions resulting in a delta OD significantly different from the one measured at the previous time point (Wilcoxon-Mann-Whitney test fdr<0.05).

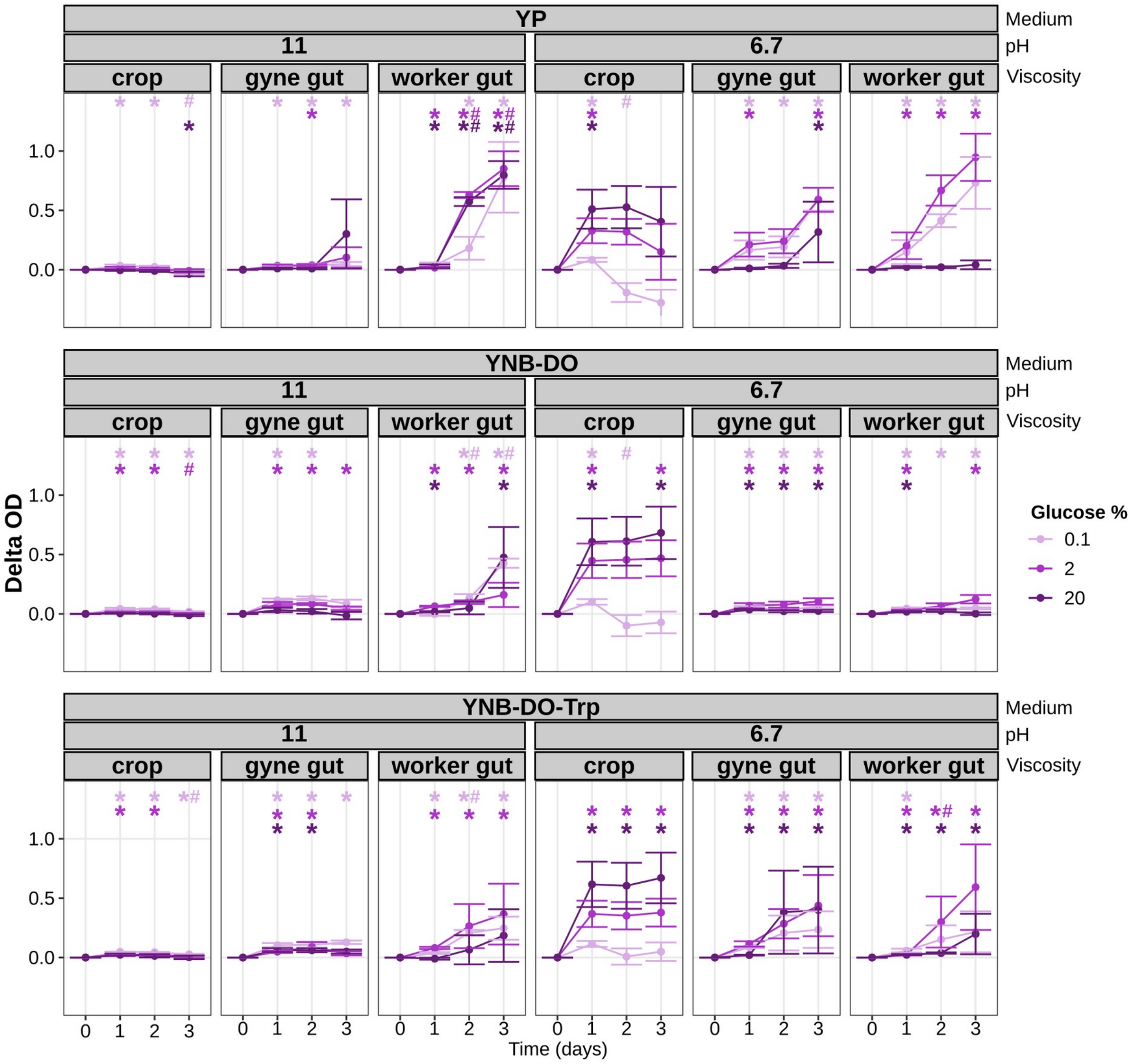

Fig. S6

Evaluation of yeast cell viability in the crop and gut environment. The data were gathered 72 hours after the cell inoculum in the tested medium. **a)** Percentage of alive cells in the tested conditions. **b)** Number of cells observed in the microscopic field in the tested conditions. **c)** Representative comparison of the number of cells and live/dead cells observed in the tested conditions; the left side ("Calcofluor White") shows the cells labeled with Calcofluor White, staining the wall of both alive and dead yeast cells; the right side ("Live/Dead test") shows the merge of the three channels: blue = calcofluor white, red= FUN1 associated with alive cells, green= FUN1 associated with dead cells. Crop - minimal medium with high concentration of tryptophan (YNB-DO+Trp), viscosity (1 mPa*s), pH 7, and intermediate glucose (2%); gut: minimal medium (YNB-DO), 52 mPa*s viscosity, 20% glucose, pH 11; YPD = rich medium, low viscosity (0.9 mPa*s), pH 7, intermediate glucose (2%).

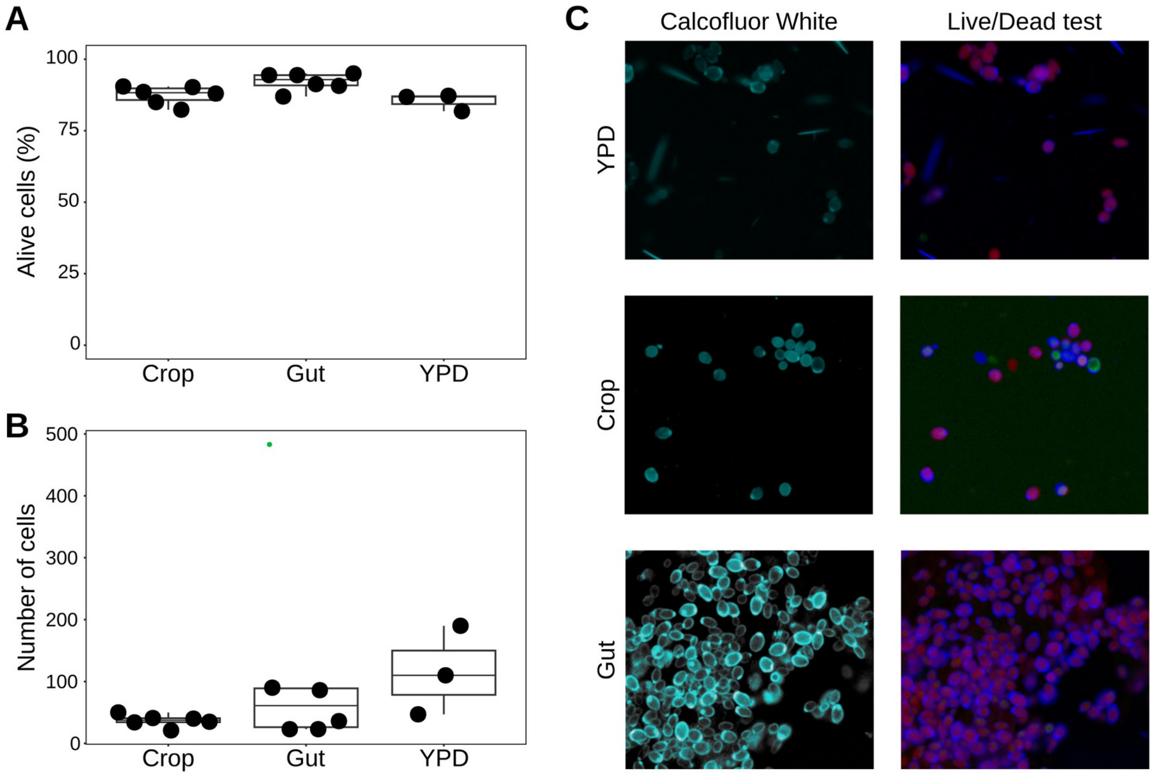

Fig. S7

Germination and growth of yeast spores in environmental conditions mimicking the wasp intestine. The plots indicate the average delta OD (obtained as the difference between the culture OD measured at the indicated time point and the OD of the same culture measured at the moment of inoculation) and standard deviation (error bars) of three biological replicates. Medium, pH, and viscosity are indicated on top of each plot; glucose is indicated as shown in the color legend. Asterisks indicate conditions resulting in a delta OD significantly different from the one measured at the inoculation time, with the color corresponding to the concentration of glucose; hashtags indicate conditions resulting in a delta OD significantly different from the one measured at the previous time point (Wilcoxon-Mann-Whitney test fdr<0.05).

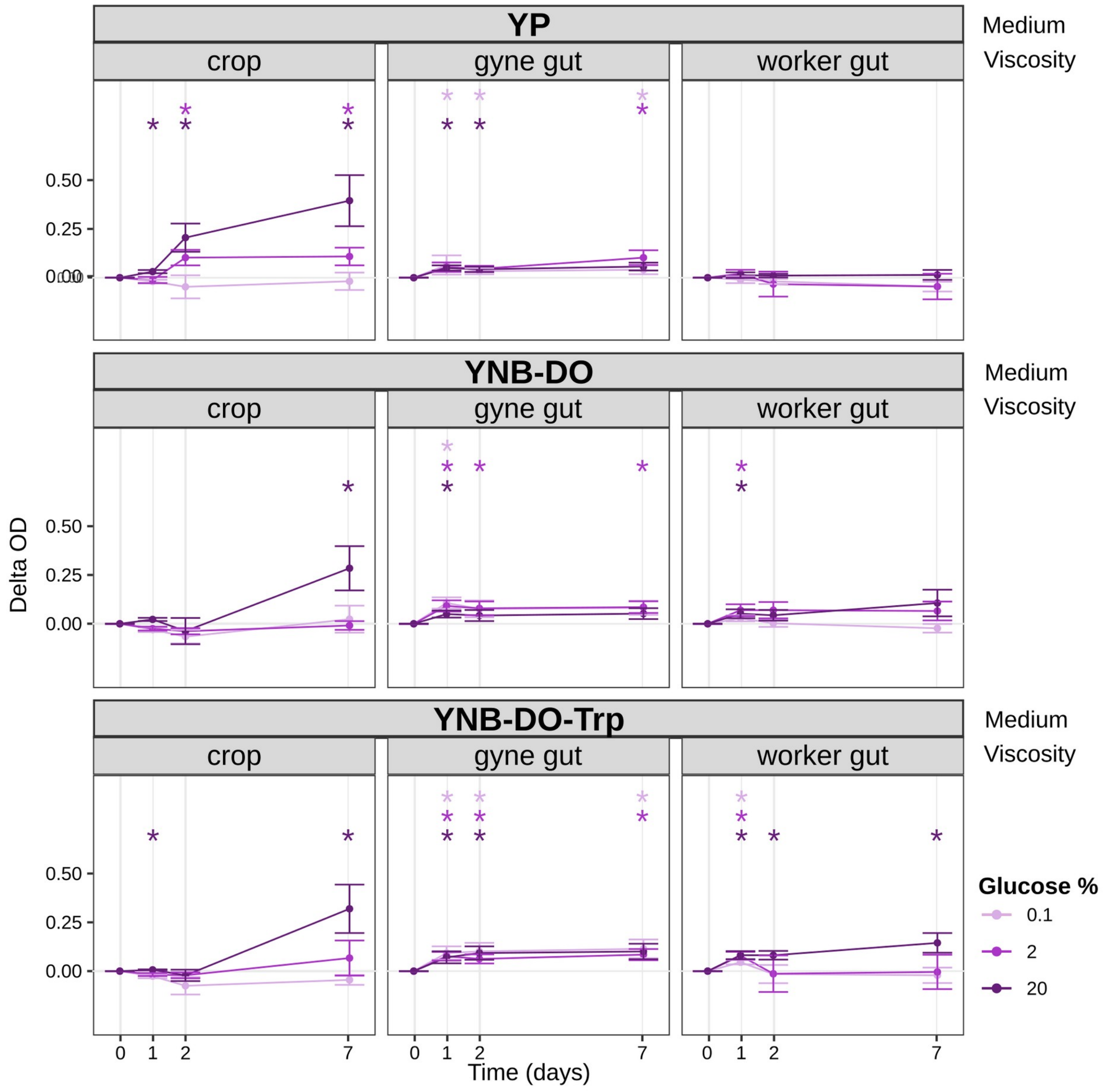

Supplement: Supplementary_materials_last_wraf243 [file supplementary_materials_last_wraf243.pdf]
